# Epidemiology of neonatal jaundice at Punakha District Hospital, Punakha, Bhutan

**DOI:** 10.1093/inthealth/ihac077

**Published:** 2022-12-05

**Authors:** Nima Dorji, Manish Raj Gurung, Dawa Gyeltshen, Krishna Singh Mongar, Sonam Wangmo, Tsheten

**Affiliations:** Punakha District Hospital, Punakha, Bhutan; Punakha District Hospital, Punakha, Bhutan; Royal Center for Disease Control, Thimphu, Bhutan; Australian National University; Punakha District Hospital, Punakha, Bhutan; Punakha District Hospital, Punakha, Bhutan; Royal Center for Disease Control, Thimphu, Bhutan; Australian National University

**Keywords:** Bhutan, epidemiology, neonatal jaundice, risk factors

## Abstract

**Background:**

Although neonatal jaundice is a global health issue, the epidemiology of this disorder remains poorly understood in Bhutan. This study aimed to examine the prevalence and risk factors of neonatal jaundice among neonates in Punakha District Hospital, Punakha, Bhutan.

**Methods:**

This cross-sectional study included 180 neonate–mother pairs registered/admitted to Punakha District Hospital between 2019 and 2020 by employing a population-based sampling technique. The data were collected using a questionnaire developed by the researchers. Univariable and multivariable logistic regression was employed to identify factors associated with neonatal jaundice.

**Results:**

The prevalence of neonatal jaundice was 33% and 47% in 2019 and 2020, respectively. Medical problems during pregnancy (adjusted odds ratio [AOR] 3.33 [95% confidence interval {CI} 1.10 to 10.31]), neonatal complications (AOR 6.59 [95% CI 1.27 to 34.16]), maternal B blood group (AOR 5.22 [95% CI 1.16 to 23.50]) and maternal O blood group (AOR 2.34 [95% CI 1.03 to 5.33]) were significantly associated with neonatal jaundice. However, neonates born via caesarean section were 92% less likely to get jaundice compared with their vaginally born counterparts (AOR 0.078 [95% CI 0.01 to 0.67]).

**Conclusions:**

A high prevalence of neonatal jaundice was found in this study. Effective management of maternal medical problems during pregnancy, preventing neonatal complications, vigilant monitoring of neonates born to mothers with B and O blood groups and vaginal delivery are critical to prevent severe hyperbilirubinemia and its associated morbidity and mortality.

## Introduction

Jaundice is a yellowish discoloration of the skin and/or conjunctiva that results from the accumulation of unconjugated bilirubin in the blood.^1^ Based on the aetiology of the disease, neonatal jaundice is classified into physiological and pathological jaundice.^2^ Physiological jaundice is a mild, self-limiting adaptation process that usually occurs 36 h after birth and resolves without treatment, while a more severe form called pathological jaundice may appear within 24 h of birth^1,2^ or later depending upon the cause.^2–4^ Failure to identify and treat severe neonatal jaundice can result in life-threatening neurological complications, including bilirubin encephalopathy and kernicterus spectrum disorder.^5,6^ Factors that increase the risk for neonatal jaundice include but are not limited to acute haemolysis, preterm delivery, prolonged labour, premature rupture of the membrane, bruises and cephalohematoma, weight loss >10%, neonatal asphyxia, neonatal infection, family history of jaundice, lower gestational age, low birthweight and male gender.^1,2^

Neonatal jaundice is associated with serious neonatal health consequences, especially in resource-limited settings.^2,5^ The disease also imposes a substantial burden on the healthcare resources.^7^ It affects half of full-term newborns and 80% of preterm newborns on a global scale.^2,5^ The incidence of neonatal jaundice is reported to be highest in the African region, with 667.8 per 10 000 live births, followed by 251.3 per 10 000 in Southeast Asia.^8^ Neonatal jaundice, which appears in the early neonatal period within 6 d of life, accounts for 1309.3 deaths per 100 000 and is identified as the seventh most common cause of neonatal mortality globally,^5^ while late neonatal jaundice, which appears in days 7–30 of neonatal life, is the seventh most common cause of neonatal death in South Asia.^5^

Although the health burden associated with neonatal jaundice is evident in several countries around the world,^2,5,8^ such data are limited in Bhutan. A retrospective study carried out in the Eastern Regional Referral Hospital, Mongar, Bhutan showed that nearly half (49.5%) of the neonates admitted in the neonatal intensive care unit (NICU) had neonatal jaundice as a co-occurring morbidity, indicating high prevalence of the disease.^9^ However, this study excluded cases of neonatal jaundice in the general ward and in the outpatient department, which report a higher number of cases than the NICU. This would have resulted in major information bias in the precise understanding of the epidemiology of neonatal jaundice. Therefore the current study was conducted to assess the prevalence and risk factors of neonatal jaundice using a population-based approach by considering all neonates registered/admitted in Punakha District Hospital in 2019 and 2020.

## Methods

### Study design

This was a cross-sectional study analysing hospital records of neonatal jaundice between January 2019 and December 2020.

### Study setting

**Figure 1.**
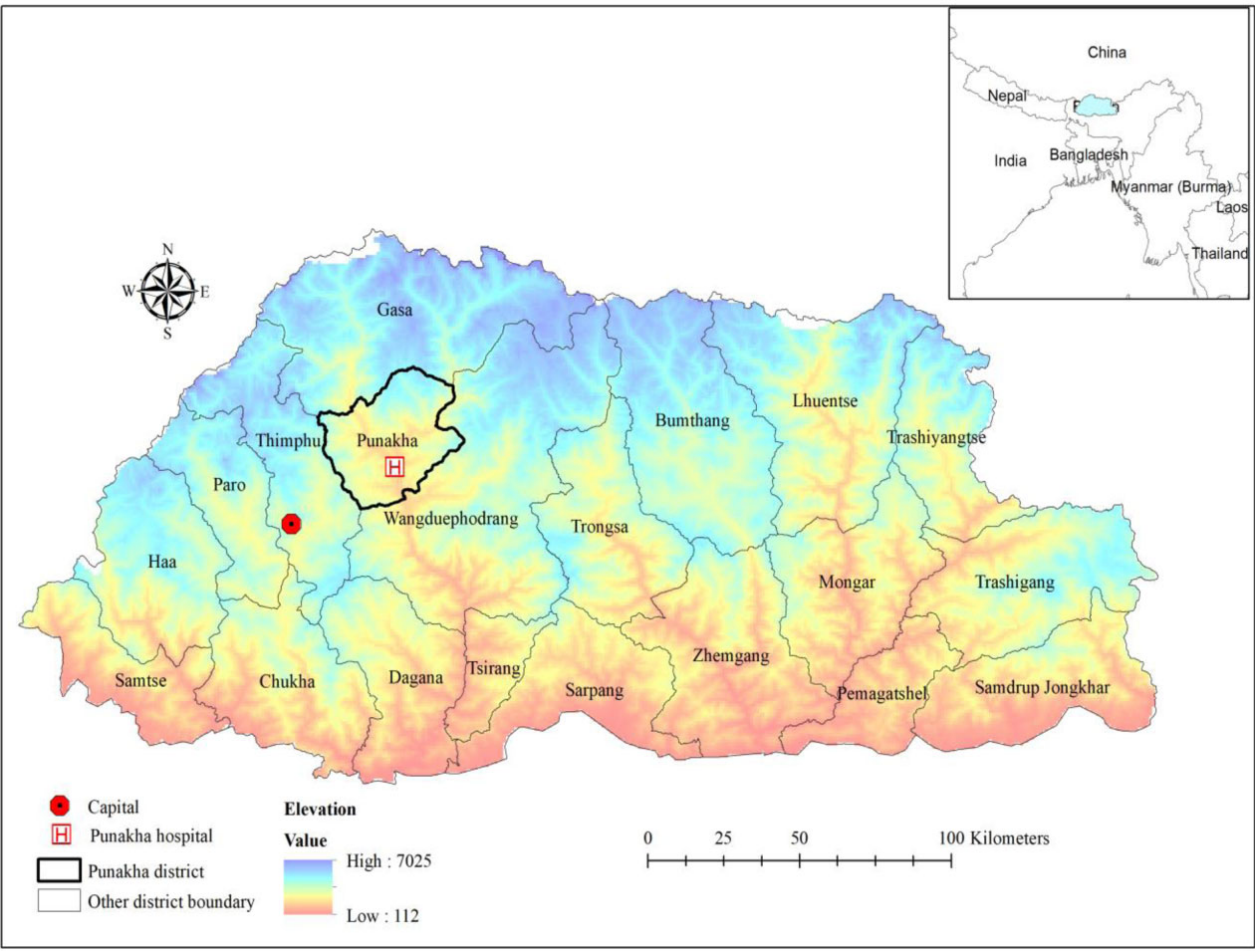
Study setting: Punakha District Hospital, Punakha, Bhutan.

The study was carried out in Punakha District Hospital, which is approximately 2-h drive from the capital city, Thimphu (Figure 1). It is a 40-bed hospital providing general medical services ranging from paediatric to geriatric cases. As per the record maintained by the hospital, the hospital saw >350 maternal–neonatal pairs in a span of 2 y (2019–2020).

Punakha District has played a pivotal role in the political and spiritual history of Bhutan. It was the capital of the country before being shifted to the current location, Thimphu, in 1907 and the first-ever national assembly was held here in 1953. Currently it is the winter residence of the central monastic body headed by His Holiness the Je Khenpo (Chief Abbot) of Bhutan.

### Case definitions

A neonate was defined as a baby <28 days old.^10^ Neonatal jaundice is a neonatal medical condition diagnosed by a physician based on clinical judgment and values of transcutaneous bilirubin. A case was any neonate diagnosed with neonatal jaundice who was hospitalized for treatment. A control was any neonate registered with the hospital but without a diagnosis of jaundice.

### Study population and sampling

The target population included all neonates whose medical records were available in Punakha District Hospital between 2019 and 2020. Neonates with incomplete or missing data were excluded from the study in the main analysis. Sample size calculation was not done, as all cases with jaundice were included in the study.

A total of 354 records of neonates were available in the study period, of which 139 were hospitalized with neonatal jaundice. To calculate the prevalence rate we used this entire dataset without excluding any missing variables. This gave a more precise estimate than computing prevalence by excluding missing variables. Complete data for 90 neonates with neonatal jaundice (cases) and 90 without the disorder (controls) were included in the main analysis.

### Data collection

Demographic and clinical information for all mother–neonate pairs registered in Punakha Hospital from 2019 to 2020 were retrieved and reviewed using a structured questionnaire developed by the researchers. The maternal register, birth register, maternal–neonatal admission and discharge forms were used as data sources.

### Statistical analysis

All variables were categorized and presented as frequencies and percentages. The association of baseline characteristics and obstetric findings with neonatal jaundice was initially assessed using univariable logistic regression. Variables with a p-value <0.2 in the univariable analysis were then introduced into the multiple logistic regression with the backward stepwise method to explore variables that were independently associated with neonatal jaundice. A p-value <0.05 was considered to be statistically significant in the final model. All analyses were done with SPSS version 21 (IBM, Armonk, NY, USA).

## Results

In 2019, 63 of 192 neonates were diagnosed with jaundice, while 76 of 162 were diagnosed with jaundice in 2020. The prevalence of neonatal jaundice was 33% and 47% in 2019 and 2020, respectively.

The majority of mothers were in the age bracket of 25–32 y (42.8%) (Table 1). Almost two-thirds (65.6%) of the mothers had primary and secondary education, 32.2% had O positive blood group, slightly more than two-thirds (66.7%) were multiparous and more than three-quarters (88.3%) did not have any medical problems during pregnancy. More than two-thirds (69.4%) had attended antenatal care visits of 8–10 times and 16.7% had labour complications. In terms of gestation, 92.2% had given birth within the normal gestation period and vaginal delivery was the most common mode of delivery (92.8%).

**Table 1.** Maternal and obstetric characteristics of neonatal jaundice at Punakha District Hospital, 2019–2020

Variables		Categories	Total, n (%)	Cases, n (%)	Controls, n (%)
Age group (years)					
	16–24		63 (35.0)	29 (32.2)	34 (37.8)
	25–32		77 (42.8)	40 (44.4)	37 (41.1)
	33–41		40 (22.2)	21 (23.3)	19 (21.1)
Qualification					
	None		42 (23.3)	17 (18.9)	25 (27.8)
	Non-formal		11 (6.1)	5 (5.6)	6 (6.7)
	Primary and secondary schools		118 (65.6)	65 (72.2)	53 (58.9)
	Diploma and degree		9 (5.0)	3 (3.3)	6 (6.7)
Blood group					
	A		57 (31.7)	23 (25.6)	34 (37.8)
	B		55 (30.6)	27 (30.0)	28 (31.1)
	AB		10 (5.6)	7 (7.8)	3 (3.3)
	O		58 (32.2)	33 (36.7)	25 (27.8)
Parity (P)					
	Primipara (P1)		50 (27.8)	29 (32.2)	21 (23.3)
	Multipara (P2–P4)		120 (66.7)	57 (63.3)	63 (70.0)
	Grant multipara (>P5)		10 (5.6)	4 (4.4)	6 (6.7)
Medical problems during pregnancy					
	No		159 (88.3)	74 (82.2)	85 (94.4)
	Yes		21 (11.7)	16 (17.8)	5 (5.6)
Antenatal care visits					
	1–7		55 (30.6)	27 (30.0)	28 (31.1)
	8–10		125 (69.4)	63 (70.0)	62 (68.9)
Labour complication					
	No		150 (83.3)	70 (77.8)	80 (88.9)
	Yes		30 (16.7)	20 (22.2)	10 (11.1)
Gestation					
	Normal		166 (92.2)	84 (93.3)	82 (91.1)
	Preterm		11 (6.1)	4 (4.4)	7 (7.8)
	Post-dated		3 (1.7)	2 (2.2)	1 (1.1)
Delivery mode					
	Vaginal		167 (92.8)	78 (86.7)	89 (98.9)
	Caesarean		13 (7.2)	12 (13.3)	1 (1.1)

In terms of neonatal characteristics, more than half were female in both the case and control groups (Table 2). The majority (90.0%) of neonates in both groups had a normal birthweight.

**Table 2.** Characteristics of neonatal jaundice in Punakha District Hospital, 2019–2020

Variables	Categories	Total, n (%)	Cases, n (%)	Controls, n (%)
Sex				
	Male	81 (45.0)	39 (43.3)	42 (46.7)
	Female	99 (55.0)	51 (56.7)	48 (53.3)
Weight				
	Normal	163 (90.6)	82 (91.1)	81 (90.0)
	Low	8 (4.4)	2 (2.2)	6 (6.7)
	High	9 (5.0)	6 (6.7)	3 (3.3)

Univariable analysis showed that neonatal jaundice was significantly associated with medical problems during pregnancy, labour complications and neonatal complications (p<0.05) (Table 3). Neonates born via caesarean section were 93% less likely to develop jaundice compared with those born via the vagina. Maternal and obstetric characteristics of maternal age, educational qualifications, blood group, parity, antenatal care visits and gestation were not significantly associated with the disorder. Similarly, there was no significant association between neonatal jaundice, gender of the neonate and neonatal birthweight.

**Table 3.** Univariable analysis of the association between maternal, obstetric and neonatal characteristics of neonatal jaundice at Punakha District Hospital, 2019–2020

Variables	Categories	Odds ratio	95% CI	p-Value
Age groups (years)				
	16–24	Ref		
	25–32	1.27	0.65 to 2.47	0.49
	33–41	1.30	0.59 to 2.87	0.52
Qualification				
	Diploma or degree	Ref		
	None	1.36	0.30 to 6.20	0.691
	Non-formal	1.67	0.27 to 10.33	0.583
	Primary and secondary school	2.45	0.59 to 10.28	0.22
Blood group				
	A	Ref		
	B	3.45	0.81 to 14.73	0.095
	AB	1.42	0.67 to 3.01	0.353
	O	1.95	0.93 to 4.10	0.077
Parity (P)				
	Grant multipara (>P5)	Ref		
	Primipara (P1)	2.07	0.52 to 8.27	0.302
	Multipara (P2–P4)	1.36	0.36 to 5.05	0.649
Medical problems during pregnancy				
	No	Ref		
	Yes	3.68	1.28 to 10.52	0.015
Antenatal care visits				
	8–10	Ref		
	1–7	1.05	0.56 to 1.99	0.871
Labour complications				
	No	Ref		
	Yes	2.29	1.01 to 5.21	0.049
Gestation				
	Normal	Ref		
	Preterm	0.48	0.14 to 1.64	0.24
	Post-term	0.95	0.13 to 6.92	0.962
Delivery mode				
	Vaginal	Ref		
	Caesarean	0.070	0.01 to 57	0.013
Sex				
	Male	Ref		
	Female	1.14	0.64 to 2.06	0.653
Weight				
	Normal	Ref		
	Low	0.33	0.07 to 1.68	0.181
	High	1.98	0.48 to 8.17	0.347
Neonatal complications				
	No	Ref		
	Yes	5.5	1.17 to 25.86	0.031

Multiple logistic regression revealed that neonates born to a mother with medical problems during pregnancy were 3-fold more likely to suffer from neonatal jaundice compared with those without medical problems (Table 4). Neonates delivered via caesarean section were 92% less likely to get jaundice than those delivered vaginally, and those who sustained neonatal complications were 6.5 times more likely to suffer from neonatal jaundice. As compared with blood group A, neonates born to blood group B and O positive mothers were 5-fold and 2-fold more likely to suffer from neonatal jaundice, respectively.

**Table 4.** Backward multiple logistic regressions of maternal and neonatal characteristics predicting jaundice in neonates at Punakha District Hospital, 2019–2020

Variables	Categories	OR	95% CI	p-Value
Medical problems during pregnancy				
	No	Ref		
	Yes	3.33	1.10 to 10.31	0.037
Delivery mode				
	Vaginal	Ref		
	Caesarean	0.08	0.01 to 0.67	0.02
Neonatal complications				
	No	Ref		
	Yes	6.59	1.27 to 34.16	0.025
Blood group				
	A	Ref		
	AB	1.94	0.85 to 4.43	0.11
	B	5.22	1.16 to 23.50	0.031
	O	2.34	1.03 to 5.33	0.042

## Discussion

This retrospective study aimed to assess the prevalence of neonatal jaundice and associated factors among neonates registered in Punakha District Hospital. The prevalence of neonatal jaundice was 33% and 47% in 2019 and 2020, respectively. Neonatal jaundice was significantly associated with medical problems in mothers during pregnancy, neonatal complications and maternal B and O blood groups, while neonates born via caesarean section were less likely to develop neonatal jaundice.

The study showed a sharp rise in the prevalence of neonatal jaundice by more than 10% in 2020 compared with 2019. This could be due to the nationwide lockdown and stringent restrictions imposed in the country to battle against covid-19 pandemic in 2020, which mandated compulsory institutionalization of neonates suffering from neonatal jaundice. Thus it is probable that neonates diagnosed with jaundice who did not require close monitoring might have had outpatient treatment in 2019, which is more likely to remain undocumented in the hospital register.

A marked variation in the prevalence of neonatal jaundice was noted in the present study compared with that reported in Ethiopia,^11^ Nepal,^12^ South Africa,^13^ Egypt^14^ and Norway.^1^ This variation could be due to differences in socio-economic and cultural factors, the level of obstetric care and the study setting.^8,11^

It can also be attributable to differences in research methodology adopted in the current and previous studies.

Medical problems in mothers during pregnancy had a significant impact on the development of neonatal jaundice in the present study. The odds of developing neonatal jaundice were nearly four times higher among neonates born to mothers with medical problems during pregnancy compared with their counterparts. This is in agreement with previous studies that demonstrated a significant association between medical problems during pregnancy and neonatal jaundice.^1,15^ Maternal medical problems during pregnancy, such as diabetes and hypertension, trigger premature delivery, which is associated with neonatal jaundice due to immature liver development and high red blood cell counts.^16^

The present study showed that those neonates born via caesarean section were less likely to develop neonatal jaundice compared with their vaginally born counterparts. This is in contrast with findings reported by Yu et al.^15^ and Hansen,^1^ while it is in agreement with findings reported by Brits et al.^13^ However, other studies demonstrated an absence of any significant association between the mode of delivery and neonatal jaundice.^17–20^ The possible reason for a lower prevalence of neonatal jaundice in those neonates born via caesarean section could be due to the fact that unlike vaginally borne neonates, they are free from birth trauma, including bruising and cephalohematoma, which increases the risk for neonatal jaundice.^13,21,22^

The odds of developing neonatal jaundice were significantly higher among those neonates who sustained neonatal complications compared with those without complications. The finding of the study is consistent with previous studies that demonstrated a significant association between neonatal jaundice and neonatal complications.^1,21,23^ Neonatal complications such as cephalohematoma, birth asphyxia, meconium-stained amniotic fluid, neonatal hypothermia and neonatal sepsis have been shown to increase the risk of neonatal jaundice.^21,23^ While birth asphyxia is reported to inhibit conjugation of bilirubin by the liver, neonatal sepsis has been demonstrated to increase the risk of haemolysis of red blood cells and hepatic dysfunction, which ultimately results in neonatal jaundice.^21^

Neonates born to mothers with blood group O and B positive were at a greater risk for developing neonatal jaundice compared with those born to mothers with other blood groups. Previous studies have reported different findings on the effect of maternal blood groups in neonatal jaundice. Some demonstrated that neonates born to blood group O positive mothers were at significantly higher risk of suffering from neonatal jaundice compared with those born to mothers with other blood groups,^11,22,24^ while others did not find any significant relationship.^25,26^ A mother with blood group O positive produces anti-A and anti-B antibodies that cross the placental barrier and destroy neonatal red blood cells containing A, B or AB antigens,^19,22^ which ultimately results in jaundice. A significant association between maternal blood group B positive and neonatal jaundice is a unique and novel finding in the current study. This unique finding perhaps indicates a shift in the epidemiological characteristics of neonatal jaundice and highlights the importance of recognizing the vulnerability of neonates born to mothers with B blood group as well. Further studies comprising large sample sizes are required to substantiate this finding.

Our study is limited by its cross-sectional design, single study setting and retrospective data. In addition, since data on glucose-6-phosphate dehydrogenase, Rhesus factor and Coombs tests were unavailable, it was not possible to conclude the probable influence of these factors on neonatal jaundice in the current study. However, to the best of our knowledge, our study is the first of its kind to provide data on the epidemiology of neonatal jaundice in Bhutan.

## Conclusions

A high prevalence of neonatal jaundice was found in this study. Our findings indicated that medical problems during pregnancy, neonatal complications and neonates born via vaginal delivery and to mothers with B and O blood groups were associated with an increased risk of neonatal jaundice. This information will be of paramount importance for clinicians to monitor and improve care for neonatal jaundice.

## Data Availability

Datasets used for the study are not publicly available for ethical reasons. However, it will be available from the corresponding author upon reasonable request.
